# Rare Complication of Spinal Anesthesia, Acute Subdural Hemorrhage: Case Report

**DOI:** 10.1002/ccr3.72344

**Published:** 2026-04-03

**Authors:** Eyob Asefa Belay, Diriba Kebede Merga, Abebe Bedasa Ayana, Haileyesus Asefa Abebe, Chala Takele Ayana

**Affiliations:** ^1^ Department of Obstetrics and Gynecology Nekemte Compressive Specialized Hospital Nekemte Ethiopia; ^2^ Department of Internal Medicine Nekemte Compressive Specialized Hospital Nekemte Ethiopia; ^3^ Departments of Radiology Nekemte Compressive Specialized Hospital Nekemte Ethiopia

**Keywords:** burr hole, dural puncture, spinal anesthesia, subdural hemorrhage

## Abstract

Acute subdural hemorrhage, following spinal anesthesia, is one of the rarest complication in obstetrics. Unless detected and treated early, it may lead to permanent disability and even maternal death. Post‐dural puncture headache is the commonest cause of headache after spinal anesthesia. Severe and pronged headache, refractory to common analgesia, peripheral neurologic symptoms like weakness, mentation change and speech difficulty suggests subdural hemorrhage. Head imaging with CT or MRI is diagnostic. Conservative management or surgical intervention with burr hole and craniotomy are option of management depending on clinical severity and extent of hemorrhage. We present a case of 32 years old, gravida 3 para 2 mother, admitted to hospital and cesarean section was done for indication of previous two cesarean section scar at 39 week of gestation. Spinal anesthesia, Bupivacaine 2.5 mL, was administered using number a 24‐gauge spinal needle at L3‐L4 level. After 2 h of operation, she complained acute severe occipital headache, upper extremities paresthesia and neck pain. For these compliant, we consider post‐dural puncture headache, the commonest cause of headache after spinal anesthesia, and administered her common analgesia. Despite adequate analgesia, headache worsen and developed difficulty of speech, intermittent lower extremity weakness and paresthesia and she became confused. All vital signs were stable and in normal range. On 5th post‐operative day, she was evaluated by neurosurgeon and head MRI was taken. Head MRI indicates bilateral acute subdural hemorrhage on parietooccipital area. Amount of hemorrhage was significant and decide to operate the patient emergently. Bilateral Burr hole was done. Post‐operative course was smooth. Post‐operative course was smooth. She was appointed 2 weeks later for follow‐up and her condition was stable. In conclusion, acute subdural hemorrhage is a rare post‐spinal anesthesia complication. Clinicians should consider acute subdural hemorrhage in patients with prolonged headache and neurological symptoms after spinal anesthesia. Early diagnosis and intervention are essential to prevent permanent disability.

## Key Clinical Message

Acute subdural hemorrhage following spinal anesthesia is one of the rarest and fatal complication in obstetrics. Severe and prolonged headache and peripheral neurologic symptoms suggest subdural hemorrhage than post‐dural puncture headache. Head imaging with CT or MRI is diagnostic and option of management depends on clinical and hemorrhage severity.

## 1 Introduction

Currently, spinal anesthesia is gaining popularity in obstetrics [1]. It is the safer than general anesthesia for cesarean delivery [2]. It is widely practiced throughout the world. It is associated with less maternal and fetal complications than general anesthesia [3]. Spinal anesthesia is preferred over general anesthesia as spinal anesthesia is associated with decreased maternal death by 1.7 folds [1]. It decreases hospital stay too. Despite its safety in obstetrics, spinal anesthesia is associated with intra‐operative, which are detected during operation, and post‐operative complications, which are detected and diagnosed in post‐operative period. Intra‐operative complications are post‐spinal hypotension, high spinal block, spinal anesthesia failure, and maternal death. Post‐dural puncture headache is the commonest post‐spinal complication associated with spinal anesthesia, which is reported in one‐third of cases, characterized by headache that intensifies with standing or sitting. Post‐dural puncture headache commonly resolves during the first 5 days [1]. Other symptoms of post‐dural puncture includes dizziness, photophobia and nausea [3, 4]. Acute subdural hemorrhage following spinal anesthesia is the rarest and potentially devastating complication [4, 5, 6]. It is reported in 1 in 1–1.5 million cases [1, 3]. Pathophysiology of acute subdural hemorrhage in spinal anesthesia is not well studied but hypothesized that it results of imbalance of intracranial pressure resulted from dural space puncture which causes caudal displacement of brain. Displacement of brain causes stretching and tearing of blood vessel [4]. Hypertension, trauma, intracranial vascular anomaly like aneurysm, chronic alcoholics, bleeding disorders, drugs inducing bleeding, hematologic malignancy pregnancy, and dural puncture are risk factors [5, 7]. Physiologic and hormonal changes during pregnancy may causes hypertension, liver, and hemorrhagic complication [8]. These pregnancy associated are commonly seen in patients with preeclampsia and HELLP syndrome [8]. In addition, multiple dural puncture and large hole needles are attributed to possible development of subdural hemorrhage [9]. Post‐spinal subdural hemorrhage is associated with high maternal morbidity. Severe headache and nuchal pain are the commonest symptom of subdural hemorrhage which mimics post‐dural puncture headache [9]. Similarity of symptoms of post‐dural puncture headache and subdural hemorrhage delays diagnosis of subdural hemorrhage. Neurologic symptoms like extremity weakness, altered mentation and exaggerated and prolonged headache, plegia and paresis are more associated with post‐spinal subdural hemorrhage [2, 4, 9]. CT scan and MRI imaging are diagnostic [1, 9]. Untreated subdural hemorrhages can result in permanent disability and maternal death [3, 7]. There are different options of subdural hemorrhage treatment. Conservative management with analgesia, blood patch, burr hole, and craniotomy [3]. Surgical treatment of subdural hemorrhage depends on the extent of hemorrhage and neurologic status of the patient. Treatment of subdural hemorrhage depends on the size of hematoma and neurologic symptom that develops secondary to hemorrhage effect. Small hemorrhage and non‐significant neurologic effect can be treated conservatively [8]. Large‐sized hemorrhage exceeding 10 mm, brain midline shift greater than 5 mm and significant neurologic symptoms are treated surgically [3]. Burr hole and craniotomy is adequate to evacuate the hematoma and associated with good surgical outcome.

## 2 Case Presentation

### 2.1 History

32 years old, gravida 3 para 2 mother, at 39 weeks of gestation, was admitted to maternity ward for elective cesarean section with indication of full‐term pregnancy and previous two cesarean sections. She has antenatal follow‐up at Nekemte compressive specialized hospital and it was uneventful. She has two previous cesarean sections. The first cesarean section was indicated for failed induction and the second was for declined trial of labor after cesarean section. She was given spinal anesthesia for her two cesarean sections and she had no anesthesia associated complication. Currently, she has no headache, fever, blurring of vision, or mental status alterations. She has no history of hypertension. She has no history of diabetic mellitus. She has no history of trauma or falling accident. She has no history of drug allergy.

### 2.2 Physical Examination

Vital signs were BP‐120/75 mm gh, pulse rate 86 beats per minute, respiratory rate 20 breaths per minute, and temperature‐36^o^ C centigrade. On eye examination, conjunctiva was pink and sclera was anicteric. The pertinent physical exam findings on the abdomen were term sized gravid uterus, cephalic fetal presentation, fetal heart beat was 144 beats per minute and there is a supra‐pubic transverse old surgical scar. On obstetrics ultrasound, singleton intrauterine fetus, cephalic presentation, fundal placenta, 38 weeks aggregate gestational age, estimated fetal weight was 3600 g and adequate amniotic fluid amount. On basic laboratory tests, hemoglobin was 12.7 g/dL, platelet count was 197,000/mL, blood group was O+, HBSag, and HIV tests were negative.

On January 16, 2025, cesarean section was done. She was given spinal anesthesia using a number 24‐gauge spinal needle. She was administered 2.5 mL of bupivacaine at the L3‐L4 site. Operation was uneventful. She gave birth to an active 3.5 kg female neonate. The mother was transferred to the maternity ward with post‐operative care order. After 2 h of operation, she complained of an acute severe headache, upper extremities paresthesia, and neck pain. The headache was more severe on the occipital side. For this complaint, post‐spinal puncture headache was considered, and she was given regular analgesics. She was administered 50 mg pethidine intramuscular bid and diclofenac 75 mg intramuscular tid. She was hydrated with crystalloids. Despite the management, the headache worsened and became confused and intermittently agitated. On the fifth post‐operative day, she was evaluated by a neurosurgeon. She has intermittent lower extremities weakness and paresthesia on lower limbs. She has no history of fever. On the physical examination, all vital signs were in normal range. On head examination, there was no swelling nor laceration on the head. On central nervous system examination, kerning's and brudzinski's sign were negative.

## 3 Differential Diagnosis Considered Were

- Post‐dural puncture headache
- Post‐partum preeclampsia
- Chemical meningitis
- Bacterial meningitis
- Acute stroke
- Migraine headache
- Chronic subdural hemorrhage
- Epidural hemorrhage
- Acute Subdural hemorrhage

**FIGURE 1:**
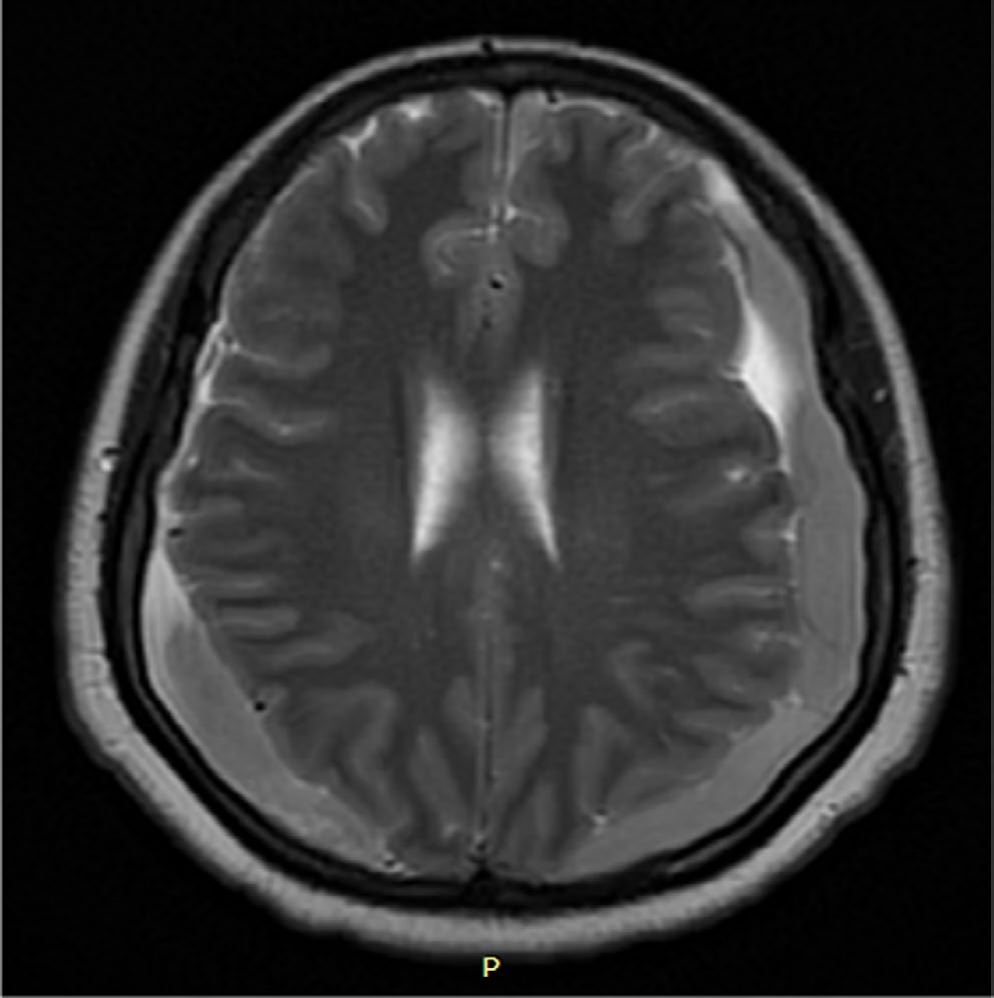
A MRI image showing right side acute subdural hemorrhage in the parietooccipital area and left hemispheric acute subdural hemorrhage.

After evaluation, she was sent for head MRI imaging. Head MRI was taken and it showed bilateral acute subdural hemorrhage Figure 1, an MRI image showing right side acute subdural hemorrhage on parietooccipital area and left hemispheric acute subdural hemorrhage was the result. The hemorrhage was significant with respect to size of hematoma and neurologic effect. She was linked to neurosurgery unit. Surgical intervention with bilateral burr was decided and she was operated.

### 3.1 Follow‐Up and Outcome

Post‐operative course was smooth. She was appointed 2 weeks later for follow‐up and her condition was stable. We believe that the medical science community will be interested in reading this article as it is a rare complication of spinal anesthesia.

## 4 Discussion

Acute subdural hemorrhage is one of the rarest and potentially devastating complications of spinal anesthesia, which are reported 1 in 1 to 1.5 million cases [1, 3]. In obstetrics, spinal anesthesia is preferred over general anesthesia as spinal anesthesia is associated with decreased maternal death by 1.7 folds [1]. Pathophysiology of acute subdural hemorrhage in spinal anesthesia is not well studied but hypothesized that it results from imbalance of intracranial pressure due to dural space puncture which causes caudal displacement of brain. Displacement of the brain causes stretching and tearing of blood vessels [1, 2]. Trauma, hypertension, bleeding disorder, intracranial vascular anomaly, chronic alcoholism, hematologic malignancy, and lumbar puncture are common risk factors [2, 7]. Physiologic and hormonal changes during pregnancy may cause hypertension, liver function derangement, and hemorrhagic complications. These pregnancy‐associated complications are commonly seen in patients with preeclampsia and HELLP syndrome [8]. In addition, multiple dural punctures and large hole needles are attributed to the possible development of acute subdural hemorrhage [9]. Severe headache and neck pain are symptoms of acute subdural hemorrhage that mimics post‐dural puncture headache. Due to the similarity of symptoms between acute subdural hemorrhage and post dural puncture headache, post‐spinal acute subdural hemorrhage diagnosis and treatment can be delayed [10]. Neurologic symptoms, like muscle weakness, speech disorder, paresis, plegia, and mentation changes are more specific to acute subdural hemorrhage [2, 11]. In our case, she developed severe headache and neck pain starting on the second post‐operative day of the cesarean section. Initially, post‐dural puncture headache was considered, and she was administered analgesia. Despite the administration of adequate analgesia, the severity of headache and neck pain worsened, and she developed intermittent lower extremity weakness. On the fifth day of the surgery, MRI was taken and showed bilateral acute subdural hemorrhage, which is indicated in Figure 1. She had no history of hypertension, trauma, or drugs induced bleeding diathesis and bleeding disorder. There were no multiple dural punctures to administer spinal anesthesia. We used spinal needle number 24, which has a smaller hole. Prior to cesarean section, she had no history of headache.

There are different options of acute subdural hemorrhage treatment. Conservative management, blood patch, burr hole and craniotomy are options of management. Surgical treatment of acute subdural hemorrhage depends on the extent of hemorrhage and neurologic status of a patient [10]. Patients with hemorrhage size exceeding 10 mm, brain midline shift greater than 5 mm and significant neurologic symptoms are treated surgically [3]. In our case, the size of hemorrhage was 10 mm × 7 mm on right side and there was hemispheric on left side. In addition, patient has peripheral neurologic symptoms like intermittent lower extremities weakness and paresthesia. She has also severe and persistent headache despite analgesia. Above mentioned reasons were adequate to intervene surgically. Bilateral burr hole was done on January 20/2025. Post‐operative course was smooth and she was discharged home on 5th post‐operative day. Two weeks later, she was evaluated and her clinical condition was stable. In general, acute subdural hemorrhage is a devastating complication of the spinal anesthesia. Clinical presentation with prolonged and severe headache, nuchal pain, peripheral neurologic symptoms suggest acute subdural hemorrhage than dural‐puncture headache. Clinical symptoms and neuroimaging with CT or MRI suggestive of acute subdural hemorrhage are diagnostic. Conservative management, blood patch, burr hole and craniotomy are the possible options of acute subdural hemorrhage management depending on the extent of hemorrhage and neurologic symptoms. Surgical intervention is recommended for larger hemorrhage and if there are peripheral neurologic symptoms. Early diagnosis and treatment of acute subdural hemorrhage following post‐spinal anesthesia is lifesaving step.

## Author Contributions

**Eyob Asefa Belay:** conceptualization, data curation, investigation, methodology, writing – original draft, writing – review and editing. **Diriba Kebede Merga:** conceptualization, methodology, writing – review and editing. **Abebe Bedasa Ayana:** conceptualization, investigation, methodology, writing – original draft. **Haileyesus Asefa Abebe:** conceptualization, writing – original draft, writing – review and editing. **Chala Takele Ayana:** conceptualization, writing – original draft, writing – review and editing.

## Funding

The authors have nothing to report.

## Ethics Statement

Written informed consent from the patient was obtained according to journal guidelines.

## Conflicts of Interest

The authors declare no conflicts of interest.

## Data Availability

Data used during this study are available with the corresponding author upon reasonable request.
